# Unusual association of primary parotid and conjunctival tuberculosis in a young immunocompetent patient: a case report

**DOI:** 10.1099/acmi.0.000982.v3

**Published:** 2025-07-10

**Authors:** Mariam Hachimi Idrissi, Qamar Zaza, Adam Benbachir, Oumaima Skalante, Soufiane Benaazza, Mohamed Allaoui, Yassine Ben Lahlou, Elmostafa Benaissa, Mariama Chadli

**Affiliations:** 1 Department of Bacteriology, Mohammed V Military Teaching Hospital, Rabat, Morocco; 2 Department of Maxillofacial Surgery, Mohammed V Military Teaching Hospital, Rabat, Morocco; 3 Department of Radiology, Mohammed V Military Teaching Hospital, Rabat, Morocco; 4 Department of Pathological Anatomy, Mohammed V Military Teaching Hospital, Rabat, Morocco

**Keywords:** conjunctiva, GeneXpert *Mycobacterium tuberculosis* (MTB)/rifampicin (RIF), parotid gland, tuberculosis

## Abstract

Extrapulmonary tuberculosis often presents as lymphadenitis. In the head and neck area, tuberculous involvement of the parotid gland is rare and challenging to diagnose due to non-specific symptoms, which can be confused with a tumour. Tuberculous conjunctivitis, although uncommon, should be considered in cases of chronic, treatment-resistant conjunctivitis. The association of primary parotid and conjunctival tuberculosis is exceptional. They are difficult to diagnose due to the non-specificity of their clinical, biological and radiological signs. We report the case of a 24-year-old immunocompetent patient with no history of tuberculosis, who presented with intermittent swelling of the left parotid gland, accompanied by unilateral redness of the eye, which appeared 2 months later, in a context of fever and night sweats. The patient also reported a foreign body sensation in his eye, mild tearing and photophobia. Clinical examination revealed painful, warm swelling of the left parotid gland, as well as localized granulomatous conjunctivitis. Cervico-parotid CT and MRI revealed two well-limited, oval, left parotid formations with a slightly thickened and enhanced wall after injection of gadolinium, suggestive *a priori* of abscessed collections. The diagnosis of primary parotid and conjunctival tuberculosis was made on the basis of histological examination of the biopsies, as well as direct examination, culture and GeneXpert. The patient progressed well on anti-bacillary treatment. Our work underlines the great importance of GeneXpert, which is a rapid and highly sensitive technique, effective in the diagnosis of extrapulmonary tuberculosis.

## Data Summary

No data were generated during this research or are required for the work to be reproduced.

## Introduction

Tuberculosis remains a significant global public health issue, with an estimated 1.7–2 billion people affected worldwide [1, 2]. This chronic, necrotizing granulomatous infection can impact any organ, although the lungs are the most commonly affected. Salivary gland involvement in tuberculosis is highly uncommon [3].

First described in 1894 by Von Stubenrauch, parotid tuberculosis is an uncommon clinical condition, with only about 200 cases documented in the literature [4]. Diagnosing it can be challenging due to its nonspecific and polymorphic clinical and radiological features, which often resemble those of a malignant tumour. It is typically associated with disseminated tuberculosis, although primary cases have also been reported [5]. Ocular tuberculosis is a rare form of extrapulmonary tuberculosis, affecting between 0.14% and 16% of individuals. It occurs due to localized lesions and inflammation resulting from haematogenous spread or an allergic reaction to a distant tuberculosis infection [1]. Any part of the eye can be affected, and there is no specific hallmark presentation. Tuberculous uveitis is the most frequent manifestation, while conjunctival involvement is rare. The first confirmed case of conjunctival tuberculosis was reported by Solmaz *et al*. [6].

The diagnosis of these two extrapulmonary localizations of tuberculosis is generally confirmed by histological examination, revealing an epithelio-giganto-cellular granuloma with caseous necrosis. However, the bacteriology laboratory plays a crucial role in the diagnosis and treatment of this pathology, thanks to specific stains such as Auramine fluorescent staining and Ziehl–Neelsen staining, as well as culture on Löwenstein–Jensen solid medium.

However, the sensitivity of direct examination is limited. Culture is specific but slow and requires a long incubation period. GeneXpert [Xpert *Mycobacterium tuberculosis* (MTB)/rifampicin (RIF) test] has become an indispensable tool in the diagnosis of extrapulmonary tuberculosis, thanks to its speed and sensitivity of around 87.5% for biopsies [7]. The principle of the Xpert MTB/RIF test is an automated *in vitro* diagnostic test using nested real-time PCR for the qualitative detection of the MTB complex and RIF resistance. The primers in this test amplify a portion of the *rpoB* gene containing the 81 bp core region. The probes are designed to differentiate between the conserved WT sequence and mutations in the core region that are associated with RIF resistance. This technique enables simultaneous detection of MTB complex and RIF resistance in less than 2 h [8].

In the PubMed online database, a single case of primary conjunctival tuberculosis associated with parotid involvement was described in 1969 [9].

We report an exceptional association of primary parotid and conjunctival tuberculosis in a 24-year-old immunocompetent patient.

## Case presentation

A 24-year-old immunocompetent patient, with no notable medical history and no known contact with tuberculosis, presented with a left parotid swelling of intermittent nature for the past 4 months, associated with unilateral redness of the same-side eye that appeared 2 months later. The patient also reported a foreign body sensation in his eye, mild tearing and photophobia. The overall condition was stable, with nocturnal sweating and undetermined fever. The patient had no known history of chronic illnesses such as diabetes, HIV infection, autoimmune diseases or malignancies, and there was no history of immunosuppression or prior tuberculosis treatment. At the time of presentation and prior to diagnosis, the patient was not on any long-term medications.

Clinical facial examination revealed a 3×2 cm subcutaneous, fluctuating mass in the left parotid region, associated with local inflammatory signs including erythema, tenderness and warmth of the overlying skin. Palpation confirmed the presence of a painful, warm and fluctuant swelling. Intraoral examination showed no purulent discharge from Stenon’s duct.

Eye examination revealed a localized granulomatous conjunctivitis, characterized by a raised yellowish conjunctival nodule on the upper palpebral conjunctiva, with no corneal involvement or inflammatory reaction in the anterior chamber. Right preauricular lymphadenopathy was also noted, mobile, tender and non-fluctuant. The clinical presentation is suggestive of Parinaud’s oculoglandular syndrome.

The other lymph nodes were free, and there was no hepatosplenomegaly. The rest of the clinical examination was unremarkable.

**Fig. 1.**
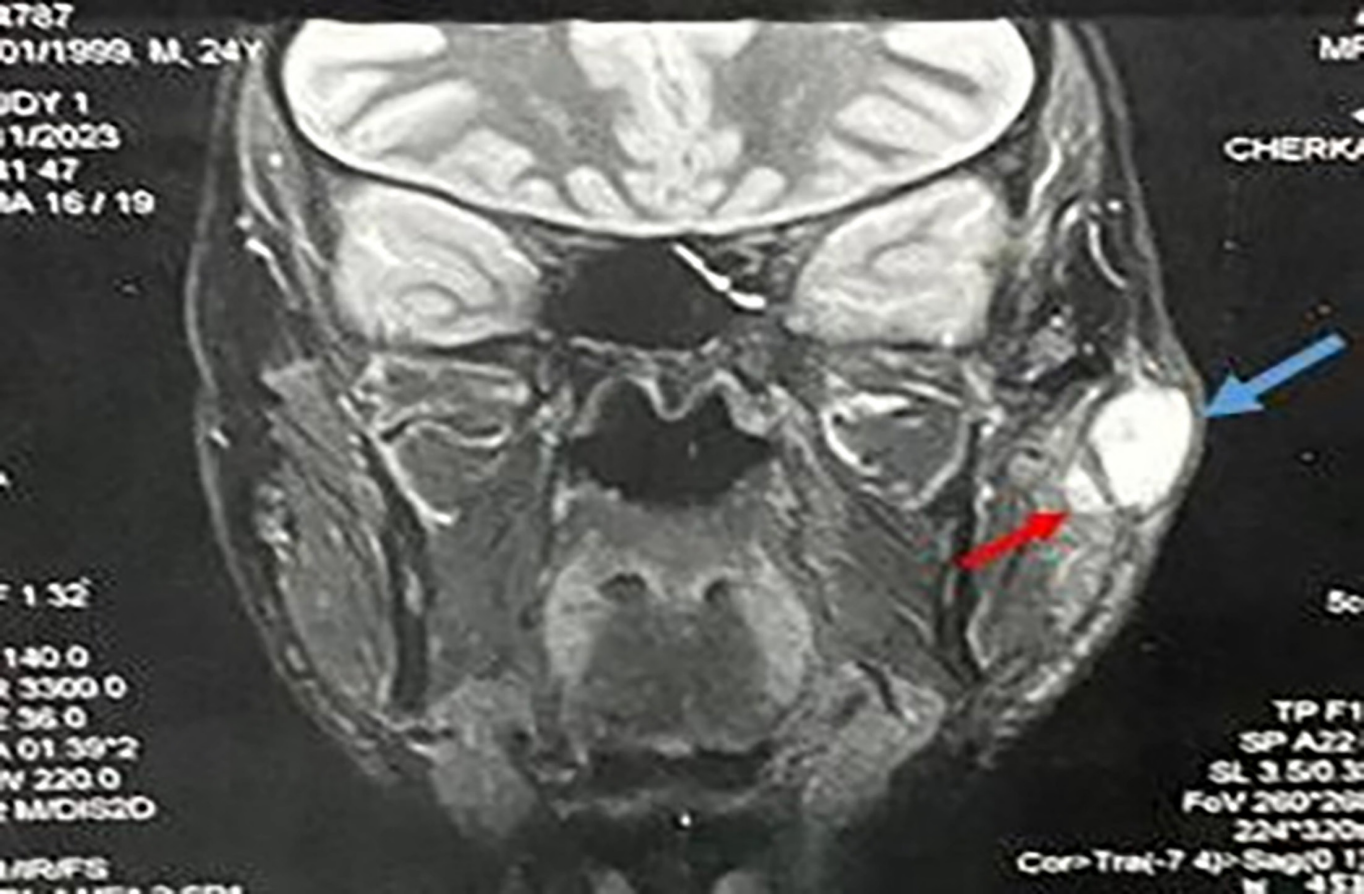
Sagittal section of cervico-parotid MRI showing two well-limited, oval, hypersignal T2, diffusion-restrictive left parotid formations, with a slightly thickened and enhanced wall after injection of gadolinium, *a priori *suggestive of abscessed collections. The first (blue arrow) measured 24×17×10 mm long axes, and the second (red arrow), inferior and medial, measured 6 mm.

Morphological examination by cervico-parotid computed tomography revealed an enlarged left parotid gland, with regular contours and a hypodense tissue nodule measuring 20 mm in diameter, slightly enhanced in the periphery after contrast. Additional cervical MRI revealed two well-limited, oval, hypersignal T2, diffusion-restrictive left parotid formations, with a slightly thickened and enhanced wall after injection of gadolinium, suggestive *a priori* of abscessed collections. The first measured 24×17×10 mm long axes, and the second, inferior and medial collection measured 6 mm, with no abnormalities in the right parotid gland (Fig. 1).

Biological tests revealed a C-reactive protein level of 7.3 mg l^−1^. Routine blood tests, including complete blood count and liver and renal function tests, were within normal limits. Serological tests were negative for hepatitis B surface antigen, hepatitis C: anti-HCV antibodies, human immunodeficiency virus (anti-HIV 1 antibodies+p24 antigen: S/CO=0.15) and syphilis. CMV IgM was negative (S/CO=0.3), while CMV IgG antibodies were positive with a high S/CO ratio (191.9), indicating past exposure to cytomegalovirus. The rest of the work-up was unremarkable. The patient was started on a course of protected amoxicillin administered intravenously, followed by oral administration for 1 month, without clinical improvement.

Given the persistence of clinical signs, drainage of the superficial abscess in the left parotid region was performed.

The specimen received by the bacteriology laboratory had a macroscopic haemopurulent appearance. On microscopic examination, Gram staining revealed a strong inflammatory reaction with a large number of neutrophils and an absence of bacterial flora.

Culture on aerobic and anaerobic media (fresh blood agar, cooked blood agar, Schaedler agar and Anaerobic Blood Agar) supplemented with nalidixic acid and colistin, as well as aerobic and anaerobic enrichment, remained sterile after 72 h incubation at 37 °C.

Given the patient’s lack of clinical improvement and the sterility of the aerobic and anaerobic cultures, the diagnosis of parotid tuberculosis was raised, and a second specimen from the same area was sent to the bacteriology and pathology laboratories.

Fluorescent staining with Auramine was performed, with positive results. This was supplemented by Ziehl–Neelsen staining.

**Fig. 2.**
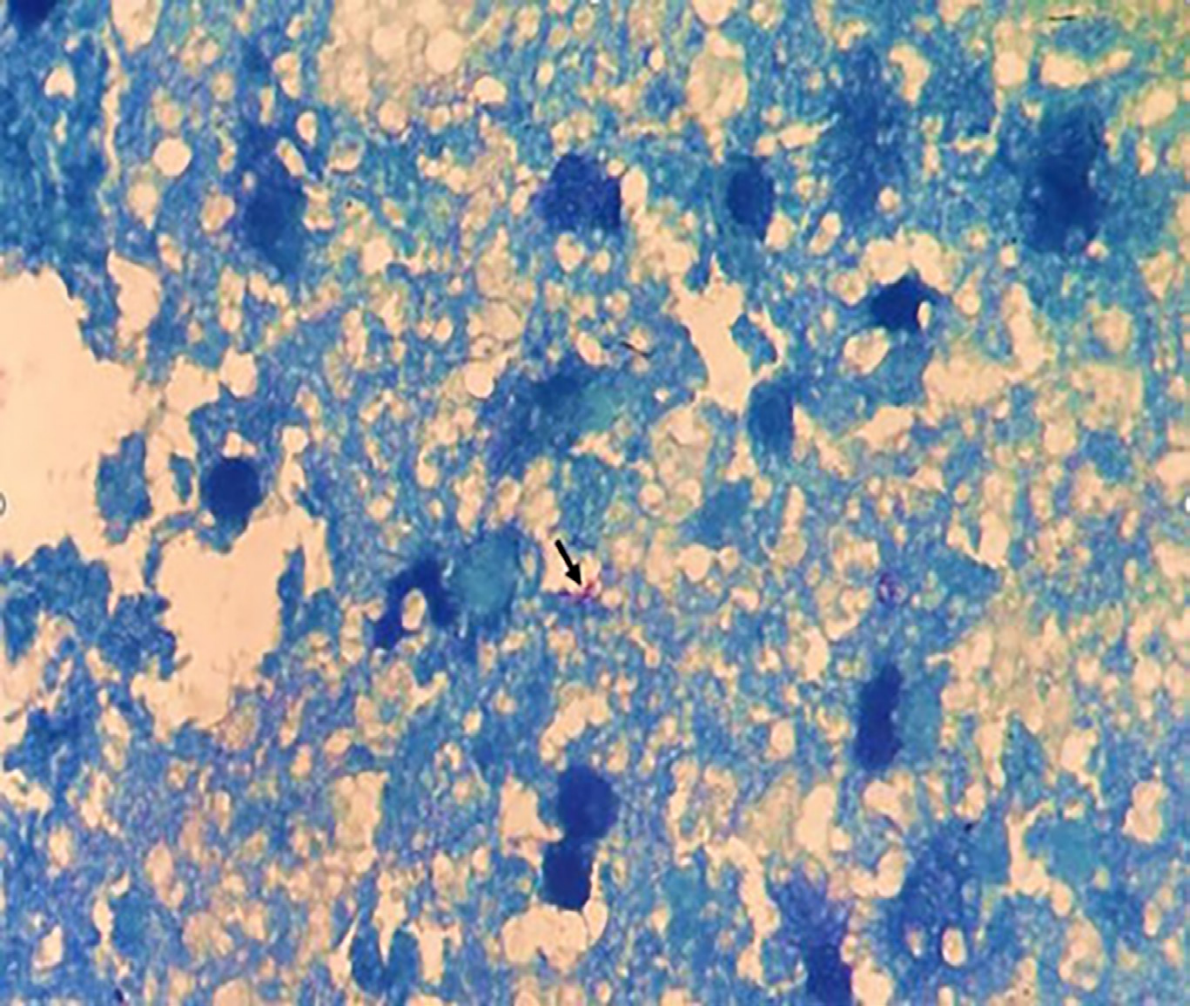
Ziehl–Neelsen staining revealing acid-fast bacilli in the parotid swab, which appear pink on a blue background (black arrow).

Careful examination of the stained slide revealed between one and ten bacilli per 300 fields (Fig. 2).

**Fig. 3.**
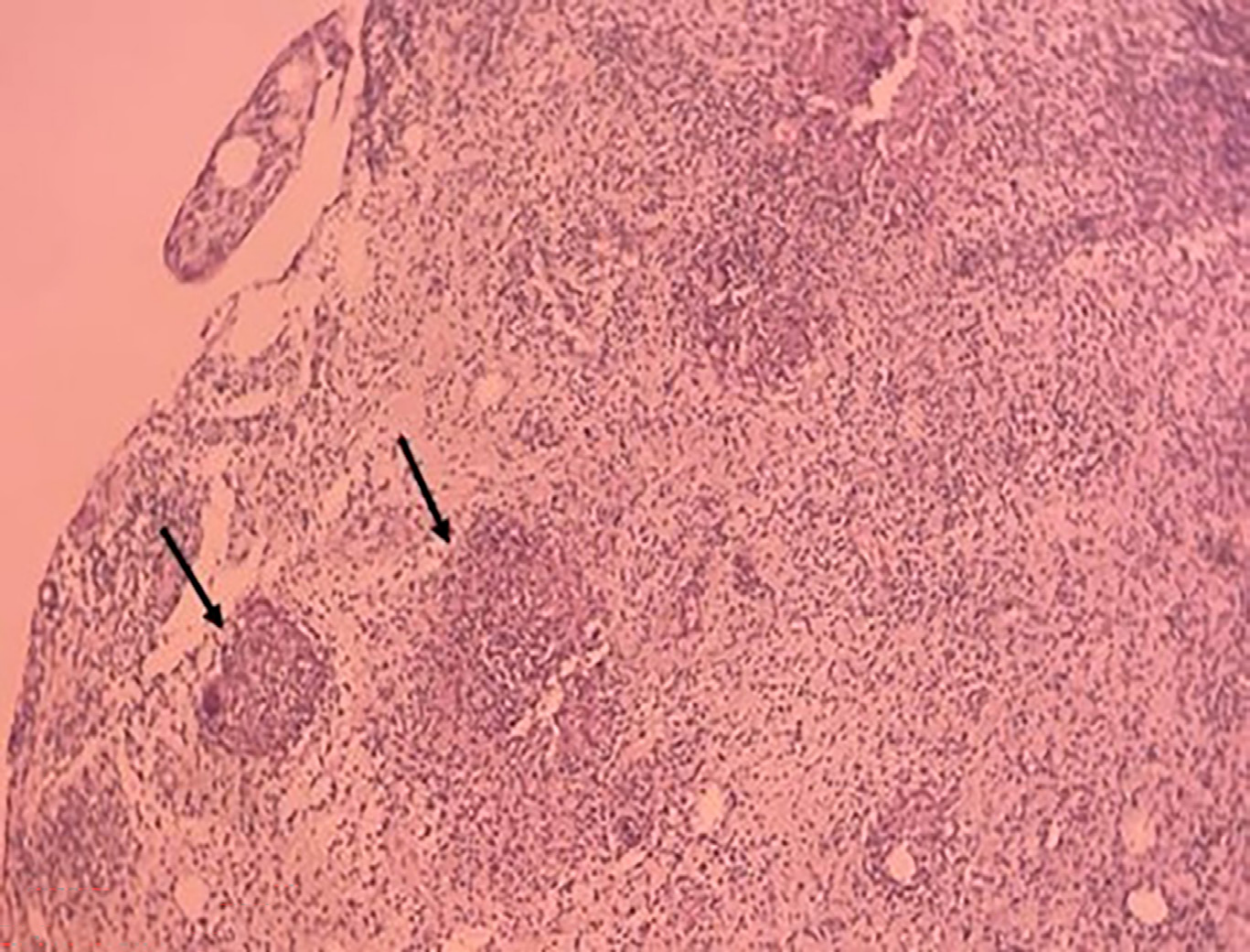
Pathological examination of a fragment of the subcutaneous swelling in the left parotid region reveals an inflammatory granulomatous reaction with numerous non-confluent epithelioid and giganto-cellular granulomas of variable size (black arrow) and no caseous necrosis.

**Fig. 4.**
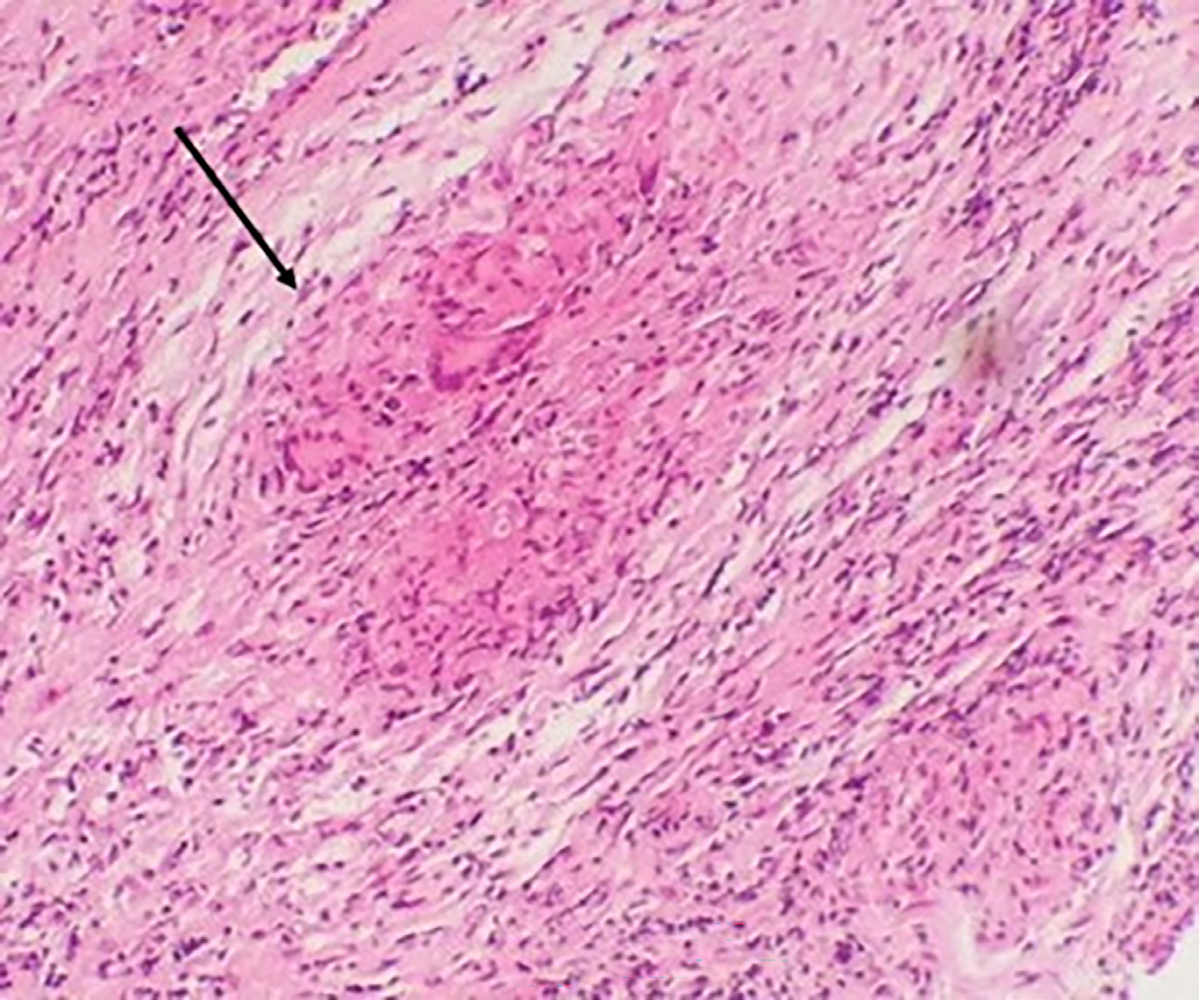
Pathological examination of the conjunctival fragment showing an abraded conjunctival lining over a fibrous chorion with a granulomatous inflammatory reaction. This granulomatous reaction is made up of epithelioid and giganto-cellular tuberculoid granulomas with very focal onset of central necrosis (black arrow).

GeneXpert (Xpert MTB/RIF test), an automated real-time PCR technique, returned positive results, confirming the diagnosis of tuberculosis, with no evidence of RIF resistance or associated mutations. Culture on Löwenstein–Jensen solid medium was also positive after 21 days, with the appearance of four cauliflower-like, creamy-beige warty colonies with a rough, dry surface. Pathological examination of a fragment of the subcutaneous swelling revealed an inflammatory granulomatous reaction consisting of numerous non-confluent epithelioid and giganto-cellular granulomas of variable size, with no caseous necrosis (Fig. 3). A fragment of conjunctiva from the left eye was also sent to the bacteriology and pathological anatomy laboratory, in view of the strong suspicion of a possible associated ocular tuberculosis. Microscopic examination using Auramine and Ziehl–Neelsen stains was negative; however, GeneXpert testing for MTB was positive, again without rifampin resistance. Culture on Löwenstein–Jensen medium was positive after 42 days. Histological analysis showed conjunctival epithelium overlying a chorion exhibiting granulomatous inflammation with granulomas composed of epithelioid and giant cells, some displaying early signs of caseous necrosis. These findings were consistent with epithelioid and giganto-cellular granulomatous conjunctivitis with incipient caseous necrosis, compatible with ocular tuberculosis (Fig. 4).

There was no associated pulmonary involvement, with a normal chest X-ray, and the MTB testing in sputum on three successive days was negative.

Medical treatment with anti-bacillary chemotherapy was started, according to the 2RHZE/4RH therapeutic protocol. This regimen includes a combination of isoniazid, RIF, ethambutol and pyrazinamide for 2 months, followed by a 4-month course of two drugs: isoniazid and RIF, for a total of 6 months of anti-tuberculosis treatment [5], with a good clinical evolution in terms of regression of the parotid swelling and improvement of his conjunctivitis, and a notable radiological improvement. Unfortunately, no post-treatment imaging is available.

## Discussion

Non-nodal tuberculosis of the ear, nose and throat sphere accounts for only 1.8% of extrapulmonary tuberculosis cases. Parotid gland tuberculosis is a very rare form of extrapulmonary tuberculosis, with less than 200 cases reported in the world literature [4]. This rarity may be explained by the presence of thiocyanate ions and proteolytic enzymes, which exert antibacterial activity, offering relative protection, while salivary flow reduces the risk of stagnation [10].

There are several hypotheses concerning the source of tuberculosis infection of the parotid gland. It has been proposed that direct inoculation of MTB may originate from local sites such as infected tonsils, the oral cavity or the external auditory canal. The infection could also spread from the injured oral mucosa of infected molar teeth. Another possible route is haematogenous dissemination, in the case of tubercular or lymphatic miliaria from a distant primary pulmonary focus.

In many reported cases, as in ours, there is no evidence of concurrent pulmonary tuberculosis, which raises the hypothesis of haematogenous, lymphatic or direct spread from a latent or undiagnosed focus. Some authors suggest that the presence of MTB in the oral cavity or upper aero-digestive tract could lead to direct inoculation into the gland via Stensen’s duct [11].

Tuberculous parotitis can take two forms: diffuse, resembling common inflammation, or localized, with involvement of intra- and peri-glandular lymph nodes [12].

This pathology, which most often affects young adults between the ages of 20–40 years, manifests itself clinically as progressive, unilateral swelling of the parotid gland, either diffuse or nodular, simulating a pseudo-tumoural syndrome. Pain, trismus and facial paralysis may also occur but are more consistent with neoplastic pathology. Signs of tuberculosis impregnation may also be present [12, 13]. Parotid tuberculosis poses a real problem of differential diagnosis, as there is no clinical, biological or radiological evidence to guide the clinician. In fact, several radiological aspects can be seen: diffuse sialadenitis, parotid tissue nodule, cyst or liquid process, more or less associated with jugulocarotid and intraparotid adenopathies. The differential diagnosis of the diffuse form is essentially carcinoma or lithiasis parotitis, while the circumscribed form is more suggestive of adenitis, a cyst or a mixed tumour [12].

From a biological point of view, an inflammatory syndrome is generally observed, and the tuberculin intradermal test is not systematically positive [12].

Fine-needle aspiration cytology, a simple procedure that carries fewer complications compared to conventional parotidectomy, is accurate in distinguishing parotid tuberculosis from neoplasms and in differentiating chronic swelling from acute suppurative parotitis [14].

Biological diagnosis of tuberculosis is based on the presence of MTB in biopsies obtained by direct examination and culture, and the presence of epithelioid and giganto-cellular granulomas with caseous necrosis on histological examination.

In cases where direct examination and culture are negative, or where samples are pauci bacillary, PCR amplification of mycobacterial DNA fragments in tissue or biopsy samples is useful for diagnosis, providing a rapid result, detection of RIF resistance and very high sensitivity, even in the early stages of the disease [6, 15].

The association of primary tuberculous parotid involvement and granulomatous conjunctivitis of tuberculous origin is exceptional, especially when the patient is young and immunocompetent, as in our case. A single case of primary conjunctival tuberculosis was described in 1969, this time associated with parotid involvement [9]. In fact, ocular involvement can originate either by haematogenous diffusion or by distant tuberculosis infection, as was the case in our patient [1]. The primary involvement in our case was considered to be of parotid origin, since it appeared first, 2 months before the conjunctival involvement.

The first confirmed case of conjunctival tuberculosis was reported by Koaster in 1873, with several additional cases documented until the early 20th century. In 1912, Eyre examined 206 cases in total, providing detailed descriptions of 24 cases and highlighting the distinct characteristics of conjunctival tuberculosis. Involvement of the conjunctiva usually occurs by direct inoculation of the micro-organism or by contagious spread. Conjunctival lesions are usually accompanied by regional lymphadenopathy, although association with pulmonary tuberculosis is rare [6].

Although Morocco is an endemic country, conjunctival tuberculosis is not systematically evoked in the presence of conjunctivitis, not to mention the clinical variations that can pose a real problem. A recent Moroccan case report by Redouan *et al*. from Mohammed VI University Hospital in Marrakech described a 6-year-old girl with palpebral tuberculosis initially misdiagnosed as a recurrent chalazion, underscoring the diagnostic challenges in such contexts [16]. Tuberculous conjunctivitis can present as nodules, ulcerations, hypertrophic granulomatous forms and pedunculated masses, depending on morphological features [6]. In our case, it presented in nodular form.

Previously treated surgically, parotid tuberculosis is now treated with anti-tuberculosis chemotherapy according to the following protocol: 2–4 months of isoniazid, RIF, pyrazinamide and ethambutol, followed by RIF and isoniazid for 6–12 months [15].

Recent case series on ocular tuberculosis have reported the use of one to four anti-tuberculosis drugs, with treatment durations varying from 6 to 18 months [17].

Our case highlights the great importance of GeneXpert MTB/RIF, a real-time PCR technique, in the diagnosis of extrapulmonary tuberculosis, without which the diagnosis of this pathology would have been delayed, particularly for tuberculous conjunctivitis whose direct examination was negative and culture positive late on. The World Health Organization has recommended this technique as the standard molecular diagnostic method for MTB [15].

## Conclusion

Primary tuberculosis of the parotid gland is very rare. The non-specific nature of its clinical and radiological manifestations makes its diagnosis difficult, as it may be mistaken for a tumoural pathology. The diagnosis of parotid tuberculosis should be emphasized, particularly in endemic countries, as it can be successfully treated by medical means without recourse to surgery [13]. In this context, the GeneXpert MTB/RIF test proves to be a valuable diagnostic tool due to its rapidity, sensitivity and ability to detect RIF resistance, thereby aiding early and accurate management.

Although tuberculous conjunctivitis is now very rare, it must be taken into account in the differential diagnosis of cases of chronic unilateral conjunctivitis resistant to treatment [6].

The association of these two forms of extrapulmonary tuberculosis with primary involvement of the parotid gland, particularly in young immunocompetent patients, is exceptional.

## Abbreviations

MTB: *Mycobacterium tuberculosis*
RIF: rifimpacin

## References

[1] Nagar S, Khan N, Bawa SS, Sharma P, Venkatesan V (2024). A detailed overview of extra pulmonary tuberculosis pathophysiology and its diagnostic methods. Cah Magellanes-NS.

[2] Handa U, Mundi I, Mohan S (2012). Nodal tuberculosis revisited: a review. J Infect Dev Ctries.

[3] Kim YH, Jeong WJ, Jung KY, Sung MW, Kim KH (2005). Diagnosis of major salivary gland tuberculosis: experience of eight cases and review of the literature. Acta Otolaryngol.

[4] Pawar A, Sahoo N, Sharma P, Rathi N (2023). A rare case of parotid tuberculosis in a pediatric patient: a case report. Liet Chir.

[5] Kallel S, Mnejja M, Ksentini A, Ayadi S, Hammami B (2017). La tuberculose de la glande parotide: diagnostic différentiel avec une tumeur maligne. JIM Sfax.

[6] Solmaz N, Önder F, Demir N, Altuntaş Aydın Ö (2018). Primary conjunctival tuberculosis. Turk J Ophthalmol.

[7] Marouane C, Smaoui S, Kammoun S, Slim L, Messadi-Akrout F (2016). Évaluation du genexpert® MTB/RIF dans la détection moléculaire de la tuberculose extra- pulmonaire et de la résistance à la rifampicine. Med Mal Infect.

[8] Pandey S, Congdon J, McInnes B, Pop A, Coulter C (2017). Evaluation of the GeneXpert MTB/RIF assay on extrapulmonary and respiratory samples other than sputum: a low burden country experience. Pathology.

[9] Salgarello G, Focosi F (1969). Tubercolosi primaria della congiuntiva con interessamento parotideo. Chir Patol Sper.

[10] Singh D, Mishra S (2021). A rare case of parotid gland tuberculosis. Case Rep Pediatr.

[11] Alotaibi M, Alqahtani A, Alshahrani A (2022). Parotid gland tuberculosis: a case report and literature review. Cureus.

[12] Dangore-Khasbage S, Bhowate RR, Degwekar SS, Bhake AS, Lohe VK (2015). Tuberculosis of parotid gland: a rare clinical entity. Pediatr Dent.

[13] Kamal D, Oufkir A, Bezzari A, Maaroufi M, El Alami MN (2015). Tuberculose primaire de la glande parotide: à propos d’un cas. Acta Odontostomatol.

[14] Yang F, Wu M, Peng Y, Dong X, Ge Y (2022). Do not ignore tuberculosis of the parotid gland when meeting obvious infiltration of neutrophils in a suspicious swelling by FNAC. Ear Nose Throat J.

[15] Zhang Q, Zhang Q, Sun B-Q, Liu C, Su A-N (2018). GeneXpert MTB/RIF for rapid diagnosis and rifampin resistance detection of endobronchial tuberculosis. Respirology.

[16] Redouan A, Hamdani H, El Maaloum L, Allali B, El Kettani A (2021). Primary palpebral tuberculosis: a case report. Eur J Med Health Sci.

[17] Alvarez GG, Roth VR, Hodge W (2009). Ocular tuberculosis: diagnostic and treatment challenges. Int J Infect Dis.

